# Glucoselysine, a unique advanced glycation end-product of the polyol pathway and its association with vascular complications in type 2 diabetes

**DOI:** 10.1016/j.jbc.2024.107479

**Published:** 2024-06-13

**Authors:** Hiroko Yamaguchi, Takeshi Matsumura, Hikari Sugawa, Naoko Niimi, Kazunori Sango, Ryoji Nagai

**Affiliations:** 1 Laboratory of Food and Regulation Biology, Graduate School of Bioscience, Tokai University, Kumamoto, Japan; 2 Department of Metabolic Medicine, Faculty of Life Sciences, Kumamoto University, Kumamoto, Japan; 3 Laboratory of Food and Regulation Biology, Department of Food and Life Science, School of Agriculture, Tokai University, Kumamoto, Japan; 4 Diabetic Neuropathy Project, Department of Diseases and Infection, Tokyo Metropolitan Institute of Medical Science, Tokyo, Japan

**Keywords:** advanced glycation end-products (AGEs), aldose reductase, biomarker, blood, diabetes, fructose, glucoselysine, glucose metabolism, hyperglycemia, polyol pathway, mass spectrometry (MS)

## Abstract

Glucoselysine (GL) is an unique advanced glycation end-product derived from fructose. The main source of fructose *in vivo* is the polyol pathway, and an increase in its activity leads to diabetic complications. Here, we aimed to demonstrate that GL can serve as an indicator of the polyol pathway activity. Additionally, we propose a novel approach for detecting GL in peripheral blood samples using liquid chromatography-tandem mass spectrometry and evaluate its clinical usefulness. We successfully circumvent interference from fructoselysine, which shares the same molecular weight as GL, by performing ultrafiltration and hydrolysis without reduction, successfully generating adequate peaks for quantification in serum. Furthermore, using immortalized aldose reductase KO mouse Schwann cells, we demonstrate that GL reflects the downstream activity of the polyol pathway and that GL produced intracellularly is released into the extracellular space. Clinical studies reveal that GL levels in patients with type 2 diabetes are significantly higher than those in healthy participants, while *N*^δ^-(5-hydro-5-methyl-4-imidazolon-2-yl)ornithine (MG-H1) levels are significantly lower. Both GL and MG-H1 show higher values among patients with vascular complications; however, GL varies more markedly than MG-H1 as well as hemoglobin A1c, fasting plasma glucose, and estimated glomerular filtration rate. Furthermore, GL remains consistently stable under various existing drug treatments for type 2 diabetes, whereas MG-H1 is impacted. To the best of our knowledge, we provide important insights in predicting diabetic complications caused by enhanced polyol pathway activity *via* assessment of GL levels in peripheral blood samples from patients.

The disruption of cellular homeostasis caused by persistent hyperglycemia is a hallmark of diabetes, ultimately resulting in widespread vascular damage and leading to multiple organ dysfunction. Vascular complications in diabetes are a leading cause of morbidity and mortality that represent a growing burden on medical institutions worldwide. Three microvascular complications—retinopathy, neuropathy, and nephropathy—and three macrovascular complications—coronary artery disease, cerebrovascular disease, and peripheral artery disease—are caused by poor blood glucose control (1). Prospective clinical studies have shown a strong relationship between blood glucose control and microvascular or macrovascular complications, in both type 1 and type 2 diabetes (2, 3, 4, 5). However, as diabetic complications do not develop solely as a result of persistent hyperglycemia, the cross-sectional evaluation of complications by assessing hyperglycemia alone is not suitable in the clinical practice.

Various factors have been implicated in the pathogenesis of vascular complications, including the involvement of advanced glycation end-products (AGEs) (6). The AGEs form as a result of nonenzymatic reactions between glucose and proteins or lipids, and play important roles in the pathogenesis of diabetic retinopathy, neuropathy, nephropathy, and cardiovascular diseases (7). For instance, *N*^ε^-(carboxymethyl)lysine (CML) is generated through the oxidation of fructoselysine (FL), an Amadori product, by the oxidation with peroxynitrite (8), and accumulates with the progression of diabetes (9), and atherosclerosis (10), as well as diabetic complications, such as nephropathy (11) and retinopathy (12). The *N*^δ^-(5-hydro-5-methyl-4-imidazolon-2-yl)ornithine (MG-H1) is an AGE that has been widely studied in age-related diseases (13, 14, 15). The MG-H1 is produced through a nonenzymatic reaction between methylglyoxal, generated by the degradation of the glycolysis intermediate dihydroxyacetone phosphate, and a side chain of Arg (16). Other endogenous sources of methylglyoxal are result from autooxidation of glucose (17, 18), degradation of glycated proteins (18), oxidation of aminoacetone in threonine catabolism (19), oxidation of acetone in ketone body metabolism (20, 21), and lipid peroxidation (21). However, since approximately 90% of methylglyoxal is generated from glycolytic intermediates (15), MG-H1 presence may act as an indicator of glycolysis. The exogenous source of methylglyoxal from a normal diet is relatively low and is usually less than 1% (22). Furthermore, MG-H1 is also involved in the development of chronic kidney disease (23) and diabetic nephropathy (24).

Recently, we identified glucoselysine (GL), as an epitope of a polyclonal antibody against a fructose-modified protein (25). The main source of fructose *in vivo* is the polyol pathway, which metabolizes excess glucose independently from the insulin-dependent pathway as an alternative to glycolysis (26). This pathway has two enzymatic steps. In the first step, aldose reductase (AR) helps reduce glucose to sorbitol, with NADPH as a coenzyme. In the subsequent step, sorbitol dehydrogenase helps oxidize sorbitol to fructose, with NAD^+^ as a coenzyme (26). The upregulation of the polyol pathway in glucose metabolism can lead to excessive accumulation of sorbitol within cells, causing osmotic stress (27), as well as in the overconsumption of coenzymes, thereby resulting in the dysfunction of the glutathione cycle, while potentially increasing hydrogen peroxide levels (28). Furthermore, an imbalance between NADPH and its oxidized form can result in the upregulation of NADPH oxidase, and mediate oxidative stress (29). These polyol pathway-induced cellular dysfunctions are considered contributing factors to diabetic retinopathy (30) and neuropathy (31). Therefore, research focusing on the polyol pathway is crucial for identifying potential predictors of vascular complications in diabetes.

The polyol pathway has been evaluated for its activity based on the concentration of sorbitol (32, 33), and previous research has been conducted concurrently, focusing on AR, which governs the conversion from glucose to sorbitol (33, 34). However, epalrestat, the only AR inhibitor clinically used in Japan, is not widely used owing to its limited effectiveness in treating diabetic neuropathy. One of the reasons for this is that the polyol pathway blockade by this AR inhibitor is insufficient. Niimi *et al.* demonstrated that the knockdown of the AR gene in mouse Schwann cells upregulates the expression of the aldo-keto reductase and aldehyde dehydrogenase superfamilies mRNA, restoring the production of sorbitol and fructose (35). Additionally, as demonstrated in our previous study, sorbitol accumulation in the lens tissue of type 1 diabetic rats does not necessarily increase with prolonged disease duration (25). In contrast, GL accumulation increases in a disease duration-dependent manner (25), suggesting that AGE, such as GL, may be more suitable as a marker for diseases associated with long-term hyperglycemia.

High accumulation of GL can be attributed to the fact that it is an AGE that is difficult to degrade, similar to MG-H1 (36). GL is formed by the reaction between fructose and the *ε*-amine of Lys (25, 37); therefore, GL is fructose-modified Lys. Conversely, FL is glucose-modified Lys, an Amadori end-product from the reaction between glucose and the *ε*-amine of Lys (16, 38). Despite their similarity, it is important to distinguish between GL and FL. We have previously demonstrated that GL can be measured separately from FL in diabetic rat lenses, by using LC-MS/MS (25). Nevertheless, a method for the measurement of GL levels in human blood has not yet been established, and is therefore, not known if GL could serve as an indicator of diabetic complications in clinical settings. Moreover, unlike CML and MG-H1, GL is not produced from sugars other than fructose (25). Nevertheless, it has not been demonstrated whether GL is produced only from fructose *in vivo*. Hence, despite GL having a unique origin from fructose and being the only AGE candidate indicative of polyol pathway activity, there is a lack of biomedical evidence linking GL with fructose and the polyol pathway (25).

In this study, we aimed to demonstrate the potential of GL to serve as a polyol-pathway-activity indicator and to propose a novel approach for assessing its clinical utility by detecting GL in peripheral blood samples using LC-MS/MS. Furthermore, we investigated the relationship between serum GL levels and factors associated with polyol pathway-related diabetic complications in patients with type 2 diabetes, with a particular focus on the potential of GL as a predictor of vascular complications in diabetes.

## Results

### Determination of GL levels in human serum

**Figure 1:**
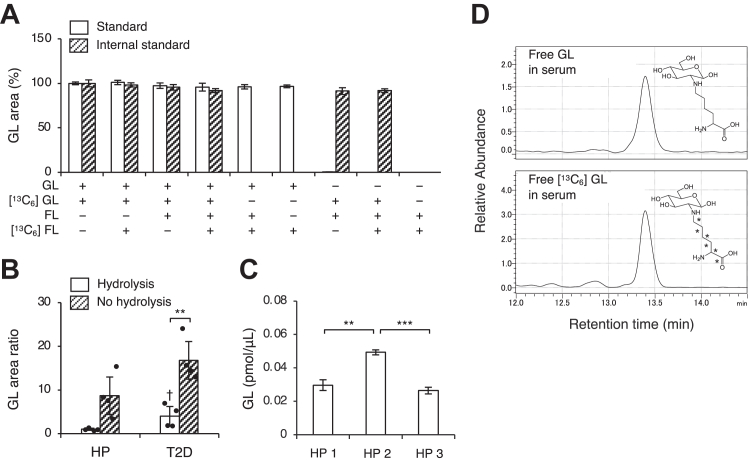
**Measuring of GL levels in human serum.***A*, GL and/or [^13^C_6_] GL were mixed with or without FL and/or [^13^C_6_] FL, and subjected to acid hydrolysis. The peak area of the GL (*open bar*) and [^13^C_6_] GL (*striped bar*) were measured using LC-MS/MS. The values in the graphs represent the mean ± SD of at least three independent experiments (*n* = 3). Dunnett test. *B*, the peak area of GL in the serum of healthy participants and patients with type 2 diabetes changed with (*open bar*) and without (*striped bar*) acid hydrolysis. The values in the graphs represent the mean ± SD of four healthy participants, and four patients with type 2 diabetes. Individual data points were represented by *closed circles*. HP, healthy participants; T2D, patients with type 2 diabetes. The significance between hydrolysis and no hydrolysis is indicated with *asterisk* (paired *t* test), and the significance between healthy participants and patients with type 2 diabetes is indicated with *dagger* (Welch's two-sample *t* test). ∗*p* < 0.05, ∗∗*p* < 0.01, ^†^*p* < 0.05. *C*, quantification of GL in the serum of healthy participants. The values in the graphs represent the mean ± SD of at least three independent experiments (*n* = 3). One-way ANOVA with Bonferroni correction, ∗∗*p* < 0.01, ∗∗∗*p* < 0.001. *D*, representative chromatograms of GL and [^13^C_6_] GL in the serum of healthy participants, obtained by LC-MS/MS. FL, fructoselysine; GL, glucoselysine; LC-MS/MS, liquid chromatography-tandem mass spectrometry.

First, a solution containing the standards and internal standards of GL and/or FL were subjected to acid hydrolysis to confirm that only GL standards and internal standards could be detected, without the influence of FL. When the peak area of the solution containing only GL was defined as 100%, addition of FL standards and internal standards did not affect the area of GL (Fig. 1*A*). A solution containing FL alone was not detected in the GL peak area.

Next, serum samples from healthy participants and patients with type 2 diabetes were subjected to acid hydrolysis after ultrafiltration, and changes in the GL peak area ratio were evaluated. The area ratio was calculated by dividing the peak area of *m/z* 309 in GL by the peak area of *m/z* 315 in [^13^C_6_] GL. The GL peak area ratio after acid hydrolysis was significantly lower in both healthy participants and patients with type 2 diabetes than in samples that did not undergo hydrolysis (Fig. 1*B*). This method could quantify variations even among healthy participants with potentially lower GL levels (Fig. 1*C*). Furthermore, distinct peaks were observed in the chromatograms for target GL and internal standards GL in the serum samples from healthy participants (Fig. 1*D*).

### Relationship between GL and fructose levels in human serum

**Figure 2:**
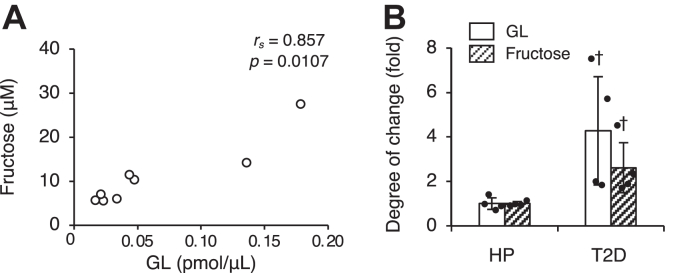
**Relationship between GL and fructose levels and the degree of changes in their levels in human serum.***A*, relationship between GL and fructose levels in human serum. *r*_*s*_, Spearman rank correlation coefficient. *B*, degree of changes in GL and fructose levels in patients with type 2 diabetes (T2D) was calculated relative to their levels in healthy participants (HP), serving as the reference. The values in the graphs represent mean ± SD of at least three independent experiments (*n* = 4). Individual data points were represented by *closed circles*. The significant difference between healthy participants and patients with T2D is indicated with a *dagger* (Mann–Whitney U test). ^†^*p* < 0.05. GL, glucoselysine.

To confirm whether GL is indeed generated from fructose *in vivo*, we measured the fructose level in the serum and investigated its correlation with GL level. There was a significantly high correlation between fructose and GL levels (*r*_*s*_ = 0.857, *p* = 0.0107) (Fig. 2*A*). Additionally, the variability between healthy individuals and patients with type 2 diabetic was 2.6-fold for fructose and 4.3-fold for GL (Fig. 2*B*).

### Involvement of polyol pathway in intracellular accumulation and extracellular release of GL in Schwann cells

**Figure 3:**
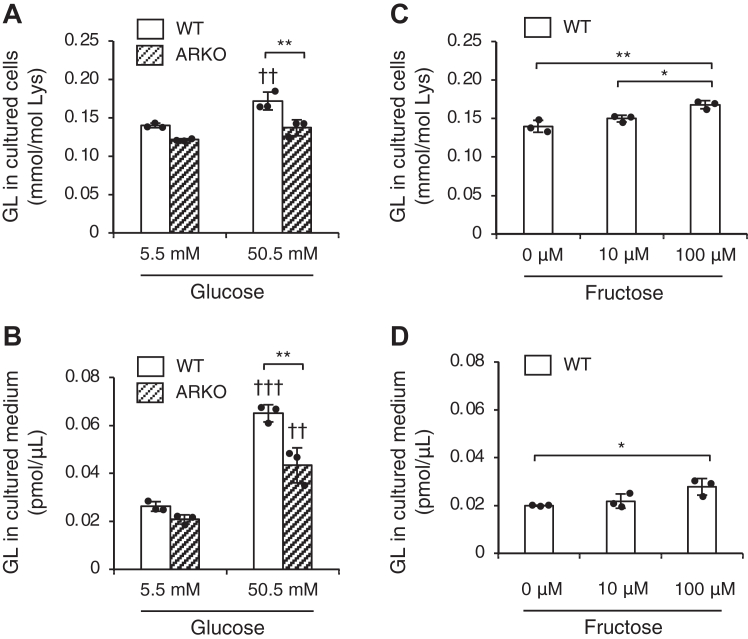
**Intracellular and extracellular GL contents under high glucose and fructose conditions.** Levels of GL in intracellular (*A*) and extracellular (*B*) compartments of WT (*open bar*) and immortalized aldose-reductase-KO (ARKO, *striped bar*) Schwann cells 72 h after exposure to normal (5.5 mM) and high glucose (50.5 mM) conditions. The values in the graphs represent the mean ± SD of at least three independent experiments (*n* = 3). Individual data points were represented by *closed circles*. The statistical significance of the difference between WT and ARKO is indicated with an *asterisk*, and the statistical significance of the difference between normal and high glucose conditions is indicated with a dagger. One-way ANOVA with Bonferroni correction, ∗∗*p* < 0.01, ^††^*p* < 0.01, ^†††^*p* < 0.001. Levels of GL in intracellular (*C*) and extracellular (*D*) compartments of WT (*open bar*) immortalized Schwann cells 72 h after exposure to 0, 10, and 100 μM fructose. The values in the graphs represent the mean ± SD of at least three independent experiments (*n* = 3). Individual data points were represented by closed circles. One-way ANOVA with Bonferroni correction, ∗*p* < 0.05, ∗∗*p* < 0.01. GL, glucoselysine.

To investigate the role of the polyol pathway in GL production, GL levels in WT and AR KO Schwann cell cultures under high sugar conditions for 3 days were compared. The results showed that GL levels intracellularly and extracellularly (in the culture medium) for AR KO cells exposed to high glucose were significantly lower compared to those for the WT (Fig. 3, *A* and *B*). However, under normal glucose conditions, no significant changes in GL levels were observed in AR KO cells compared to the WT (Fig. 3, *A* and *B*). Additionally, quantifiable levels of GL were still detected in AR KO cells under both normal and high glucose conditions (Fig. 3, *A* and *B*).

Next, we evaluated the variation in GL induced by fructose supplementation. When WT cells were supplemented with either 10 μM, equivalent to normal serum fructose levels, or 100 μM, representing high fructose levels, a significant increase in GL levels was observed compared to that seen in cells without fructose supplementation (Fig. 3, *C* and *D*). However, no dynamic variation in GL levels comparable to that induced by high glucose exposure was observed (Fig. 3, *C* and *D*).

### Comparison of GL and MG-H1 levels between healthy participants and patients with type 2 diabetes

The clinical parameters of healthy participants and patients with type 2 diabetes are shown in Table 1. The age, low-density lipoprotein cholesterol, fasting plasma glucose (FPG), and hemoglobin A1c (HbA1c) were dramatically different between the two groups (all *p* < 0.001).

**Table 1:** Clinical parameters of healthy participants and patients with type 2 diabetes

Characteristic	*n*	HP (*n* = 21)	*n*	T2D (*n* = 153)	*p* value
Age (years)	21	49 [36–52]	153	69 [61–75]	<0.001∗∗∗
Sex, male (%)	21	16 (76)	153	80 (52)	-
BMI (cm^2^/kg)	21	23.5 [20.9–24.1]	153	24.5 [22.5–27.6]	0.013∗
Current smoking (%)	18	3 (17)	152	45 (29)	-
Diabetes duration (years)	-	NA	153	11 [7–19]	NA
sBP (mmHg)	13	132 [122–141]	153	131 [120–140]	0.846
dBP (mmHg)	13	81 [75–87]	153	74 [66–79]	0.008∗∗
HDL-C (mg/dl)	14	58 [51–74]	153	52 [43–62]	0.166
LDL-C (mg/dl)	14	122 [105–135]	153	89 [69–109]	<0.001∗∗∗
TG (mg/dl)	14	98 [48–135]	153	137 [91–181]	0.030∗
FPG (mg/dl)	14	92 [86–95]	153	135 [116–171]	<0.001∗∗∗
HbA1c (%)	14	5.2 [5.1–5.3]	153	7.2 [6.8–7.9]	<0.001∗∗∗
HOMA-IR	-	NA	121	2.03 [1.48–3.06]	NA
CPI	-	NA	135	1.33 [0.95–1.73]	NA
eGFR (mL/min/1.73 m^2^)	12	71.1 [66.5–77.9]	153	66.6 [56.0–80.2]	0.083
hsCRP (mg/dl)	-	NA	151	0.08 [0.04–0.21]	NA
MICRO (%)	-	NA	153	93 (61)	NA
DR (%)	-	NA	153	41 (27)	NA
DP (%)	-	NA	153	38 (25)	NA
DN (%)	-	NA	153	66 (43)	NA
MACRO (%)	-	NA	153	38 (25)	NA
CAD (%)	-	NA	153	11 (7)	NA
CVD (%)	-	NA	153	23 (15)	NA
PAD (%)	-	NA	153	10 (7)	NA

Data are presented as median interquartile range [25–75%] with range or frequency.

BMI, body mass index; CAD, coronary artery disease; CPI, C peptide index; CVD, cerebral vascular disease; dBP, diastolic blood pressure; DN, diabetic nephropathy, DP, diabetic neuropathy; DR, diabetic retinopathy; eGFR, estimated glomerular filtration rate; FPG, fasting plasma glucose; HbA1c, hemoglobin A1c; HDL-C, high-density lipoprotein cholesterol; HOMA-IR, homeostatic model assessment-insulin resistance; HP, healthy participants; hsCRP, high sensitivity C-reactive protein; LDL-C, low-density lipoprotein cholesterol; MACRO, macrovascular complication; MICRO, microvascular complication; PAD, peripheral artery disease; sBP, systolic blood pressure; T2D, patients with type 2 diabetes; TG, triglyceride.

Welch's two-sample *t* test, ∗*p* < 0.05; ∗∗*p* < 0.01; ∗∗∗*p* < 0.001; NA, not applicable.

**Figure 4:**
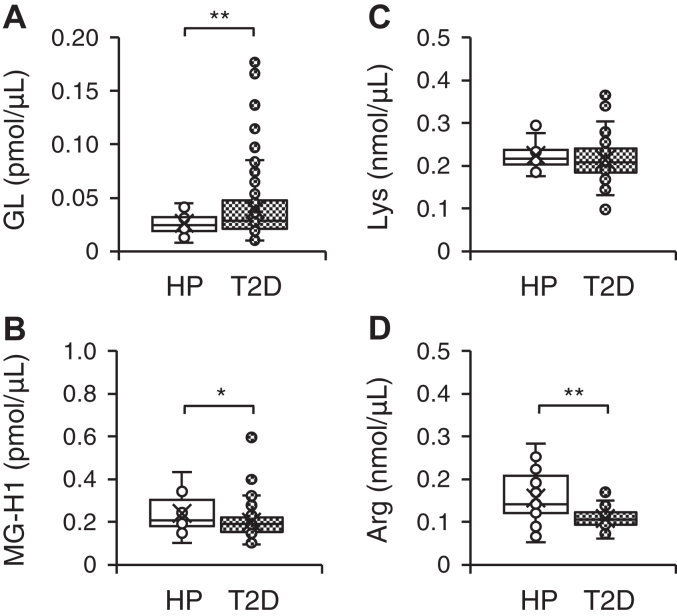
**Comparison of GL and MG-H1 levels between healthy participants and patients with type 2 diabetes.** Amounts of GL (*A*), MG-H1 (*B*), Lys (*C*), and Arg (*D*) in the serum of healthy participants (HP, *open box*, *n* = 21) and patients with type 2 diabetes (T2D, *dense dotted box*, *n* = 153). The concentration of the internal standard in the human serum was 10 pmol. Welch's two-sample *t* test, ∗*p* < 0.05, ∗∗*p* < 0.01. GL, glucoselysine; MG-H1, *N*^δ^-(5-hydro-5-methyl-4-imidazolon-2-yl)ornithine.

Differences in GL and MG-H1 levels were observed between healthy participants and patients with type 2 diabetes. The average GL levels in patients with type 2 diabetes were 1.5-fold higher than those in healthy participants (Fig. 4*A*). In contrast, MG-H1 levels were 0.8-fold lower in patients with type 2 diabetes, in relation to those of healthy participants (Fig. 4*B*). Lys levels were similar between the two groups (Fig. 4*C*), whereas Arg levels decreased by 0.7-fold (Fig. 4*D*) in patients with type 2 diabetes. In addition, the rate of change in GL levels was 34.0%, whereas the HbA1c and estimated glomerular filtration rate (eGFR) were 29.2% and −8.1%, respectively. This indicated that GL fluctuations were more pronounced when compared with those in HbA1c, an established marker of diabetes, or eGFR, an established indicator of kidney function (Table S1).

### Correlation between GL, MG-H1, and HbA1c levels and clinical parameters in type 2 diabetes

To identify clinical parameters that varied along with GL, MG-H1, and HbA1c levels, we performed a correlation analysis among patients with type 2 diabetes (Table 2, Fig. S1). Our findings showed that GL levels significantly correlated with diabetes duration (*r* = 0.384, *p* < 0.001) and eGFR (*r* = −0.444, *p* < 0.001), while MG-H1 levels significantly correlated with eGFR (*r* = −0.411, *p* < 0.001). HbA1c levels also significantly correlated with FPG (*r* = 0.434, *p* < 0.001). Multiple regression analysis revealed that diabetes duration (β = 0.353, *p* < 0.001, 95% confidence interval (CI) = 0.200/0.507) and eGFR (β = −0.417, *p* < 0.001, 95% CI = −0.575/-0.259) significantly influenced GL levels. Similarly, eGFR (β = −0.385, *p* < 0.001, 95% CI = −0.554/-0.216) independently influenced MG-H1 levels. Further, FPG (β = 0.382, *p* < 0.001, 95% CI = 0.237/0.527) independently influenced HbA1c levels (Table 2). The variance inflation factors for these indexes were all less than 2.

**Table 2:** Univariate and multivariate regression analysis assessing correlations between GL, MG-H1, or HbA1c and clinical parameters

T2D	*n*	GL		MG-H1		HbA1c		GL (*n* = 153)			MG-H1 (*n* = 153)			HbA1c (*n* = 151)		
Factor		*r*	*p*	*r*	*p*	*r*	*p*	β	*p*	95% CI (Lower/Upper	β	*p*	95% CI (Lower/Upper	β	*p*	95% CI (Lower/Upper)
Age	153	0.109	0.181	0.107	0.187	−0.105	0.195	−0.253	0.003∗∗	−0.419/−0.086	−0.141	0.120	−0.319/0.037	−0.073	0.397	−0.244/0.097
Sex, male	153	-	-	-	-	-	-	0.111	0.119	−0.029/0.250	0.040	0.600	−0.109/0.189	0.007	0.929	−0.139/0.153
BMI	153	−0.101	0.213	0.021	0.798	0.238	0.003∗∗	−0.138	0.066	−0.284/0.089	0.049	0.533	−0.107/0.206	0.132	0.079	−0.015/0.279
Duration	153	0.384	<0.001∗∗∗	0.252	0.002∗∗	0.147	0.070	0.353	<0.001∗∗∗	0.200/0.507	0.191	0.023∗	0.027/0.354	0.117	0.150	−0.043/0.277
sBP	153	−0.010	0.898	0.020	0.808	−0.020	0.811									
dBP	153	−0.110	0.177	−0.089	0.277	0.023	0.774									
HDL-C	153	−0.106	0.191	−0.055	0.498	−0.122	0.133									
LDL-C	153	0.041	0.615	0.034	0.677	−0.216	0.007∗∗							−0.121	0.126	−0.277/0.035
TG	153	0.072	0.380	0.037	0.654	0.035	0.666									
FPG	153	0.027	0.743	−4.7 × 10^−4^	0.995	0.434	<0.001∗∗∗	−0.023	0.768	−0.178/0.132	0.009	0.911	−0.156/0.175	0.382	<0.001∗∗∗	0.237/0.527
HbA1c	153	−0.065	0.423	−0.177	0.028∗	-	-	−0.031	0.699	−0.190/0.128	−0.171	0.048∗	−0.341/−0.001			
HOMA-IR	121	−0.145	0.114	0.058	0.525	0.093	0.311									
CPI	135	−0.039	0.653	0.087	0.315	0.073	0.399									
eGFR	153	−0.444	<0.001∗∗∗	−0.411	<0.001∗∗∗	0.170	0.036∗	−0.417	<0.001∗∗∗	−0.575/−0.259	−0.385	<0.001∗∗∗	−0.554/−0.216	0.216	0.008∗∗	0.058/0.373
hsCRP	151	−0.069	0.400	0.055	0.501	−0.191	0.019∗							−0.195	0.007∗∗	−0.337/−0.053

GL, *R* = 0.319, adjusted *R*^2^ = 0.286, *p* < 0.001; MG-H1, *R* = 0.224, adjusted *R*^2^ = 0.186, *p* < 0.001; HbA1c, *R* = 0.312, adjusted *R*^2^ = 0.279, *p* < 0.001. The explanatory variable of sex was of discrete type: 0 for male and one for female, while all other variables were continuous.

β, standardized partial regression coefficient; BMI, body mass index; CI, confidence interval; CPI, C peptide index; dBP, diastolic blood pressure; eGFR, estimated glomerular filtration rate; FPG, fasting plasma glucose; HbA1c, hemoglobin A1c; HDL-C, high-density lipoprotein cholesterol; HOMA-IR, homeostatic model assessment-insulin resistance; hsCRP, high sensitivity C-reactive protein; LDL-C, low-density lipoprotein cholesterol; *r*, Pearson product-moment correlation coefficient; sBP, systolic blood pressure; T2D, type 2 diabetes; TG, triglyceride.

∗*p* < 0.05; ∗∗*p* < 0.01; ∗∗∗*p* < 0.001.

### Association between vascular complications and GL, MG-H1, and HbA1c levels

**Figure 5:**
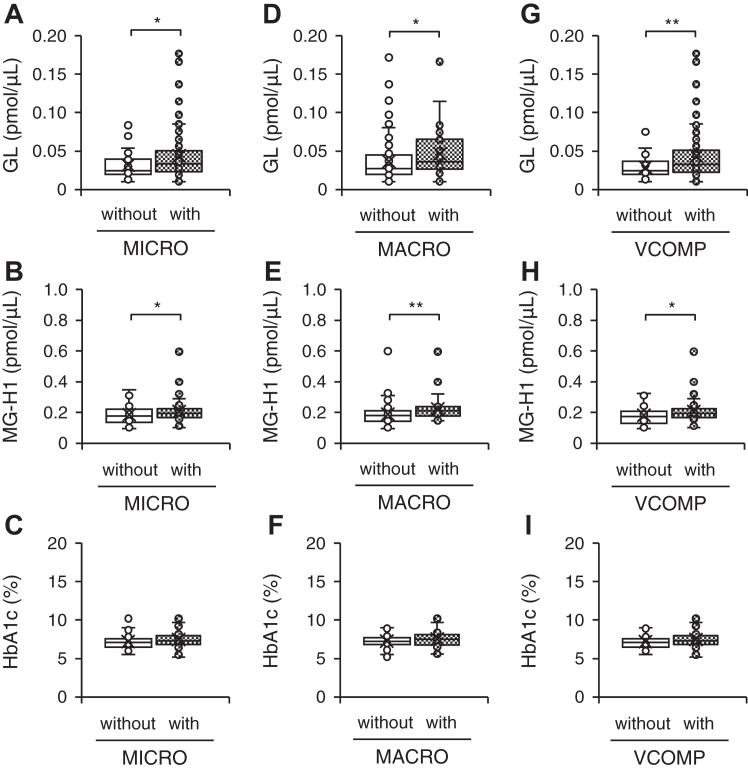
**Changes in the serum GL, MG-H1, and HbA1c levels depending on vascular complications.** Levels of GL (*A*), MG-H1 (*B*), and HbA1c (*C*) in patients with type 2 diabetes and cooccurrent microvascular complications (MICRO). Patients with type 2 diabetes without (*open box*, *n* = 60) or with (*dense dotted box*, *n* = 93) MICRO. Levels of GL (*D*), MG-H1 (*E*), and HbA1c (*F*) in patients with type 2 diabetes and cooccurrent macrovascular complications (MACRO). Patients with type 2 diabetes without (*open box*, *n* = 115) or with (*dense dotted box*, *n* = 38) MACRO. Levels of GL (*G*), MG-H1 (*H*), and HbA1c (*I*) levels in patients with type 2 diabetes and cooccurrent micro and/or macrovascular complications (VCOMP). Patients with type 2 diabetes without (*open box*, *n* = 51) or with (*dense dotted box*, *n* = 102) VCOMP. The concentration of the internal standard in the human serum was 10 pmol. Welch's two-sample *t* test, ∗*p* < 0.05, ∗∗*p* < 0.01, ∗∗∗*p* < 0.001. GL, glucoselysine; HbA1c, hemoglobin A1c; MG-H1, *N*^δ^-(5-hydro-5-methyl-4-imidazolon-2-yl)ornithine.

We next assessed the association between microvascular complications, a common symptom of type 2 diabetes, and GL, MG-H1, and HbA1c levels. Patients with type 2 diabetes with one or more microvascular complications exhibited 1.4-fold and 1.1-fold higher GL and MG-H1 levels, respectively (Fig. 5, *A* and *B*), compared with those of patients without microvascular complications. However, HbA1c levels remained relatively unaltered across patients with type 2 diabetes, regardless of microvascular complication cooccurrence or not (Fig. 5*C*). Similarly, patients with type 2 diabetes and who also presented with one or more macrovascular complications, displayed 1.3-fold and 1.2-fold higher GL, and MG-H1 levels, respectively, relative to the levels in patients without macrovascular complications (Fig. 5, *D* and *E*). The HbA1c levels were comparable across all patients with type 2 diabetes, with or without macrovascular complications (Fig. 5*F*). Additionally, patients with type 2 diabetes who also presented with one or more microvascular and/or macrovascular complications, displayed 1.5-fold and 1.1-fold higher GL and MG-H1 levels, respectively, when compared to patients without microvascular and/or macrovascular complications (Fig. 5, *G* and *H*). The HbA1c levels were comparable across all patients with type 2 diabetes, with or without microvascular and/or macrovascular complications (Fig. 5*I*). Variations in GL levels associated with these vascular complications were consistently significantly higher than those in MG-H1, HbA1c, FPG, and eGFR values (Table S1).

The binomial logistic regression analysis emphasized GL as an independent predictor of microvascular complications (odds ratio (OR) = 5.871, 95% Cl = 1.194/37.47, *p* = 0.043). By contrast, MG-H1 and HbA1c were identified as independent predictors of macrovascular complications (MG-H1; OR = 1.949, 95% CI = 1.059/3.723, *p* = 0.035 and HbA1c; OR = 1.511, 95% CI = 1.015/2.274, *p* = 0.043); however, MG-H1 was highlighted as a more suitable predictor for macrovascular complications. Moreover, GL and HbA1c were identified as independent predictors of vascular complications (GL; OR = 12.48, 95% CI = 1.838/122.9, *p* = 0.018 and HbA1c; OR = 1.524, 95% CI = 1.019/2.359, *p* = 0.048); however, GL was highlighted as a more suitable predictor for vascular complications (Table 3).

**Table 3:** Results of multivariate logistic regression for diabetic vascular complications

T2D	*n*	MICRO			MACRO			VCOMP		
Factor		OR	95% CI (Lower/Upper)	*p*	OR	95% CI (Lower/Upper)	*p*	OR	95% CI (Lower/Upper)	*p*
GL	153	5.871	1.194/37.47	0.043∗	1.220	0.285/5.084	0.784	12.48	1.838/122.9	0.018∗
MG-H1	153	1.218	0.661/2.356	0.541	1.949	1.059/3.723	0.035∗	1.439	0.729/3.022	0.314
HbA1c	153	1.381	0.951/2.055	0.098	1.511	1.015/2.274	0.043∗	1.524	1.019/2.359	0.048∗

The objective variables were discrete: 0 for patients without and 1 for patients with vascular complications. The explanatory variables were continuous variables.

CI, confidence interval; MACRO, macrovascular complications; MICRO, microvascular complications; OR, odds ratio; T2D, type 2 diabetes; VCOMP, vascular complications.

∗*p* < 0.05.

### Influence of therapeutic treatment on GL and MG-H1 levels

We evaluated the variations in GL and MG-H1 levels depending on the therapeutic drug used for treatment of patients with type 2 diabetes. In this study of randomly selected outpatients with type 2 diabetes, no patient was on AR inhibitors. Initially, a primary analysis examined to verify any changes by all therapeutic drugs, followed by a secondary analysis focused on antidiabetic drugs. As shown in Table 4, GL levels remained relatively stable between patients receiving or not the corresponding therapeutic drugs. By contrast, glucose-dependent insulin secretagogues (OR = 3.439, 95% CI = 1.263/10.040, *p* = 0.019): glucagon-like peptide-1 (GLP-1) receptor agonists (OR = 6.703, 95% CI = 1.908/25.590, *p* = 0.004) significantly elevated MG-H1 levels, whereas insulin sensitizers (OR = 0.462, 95% CI = 0.210/0.989, *p* = 0.049): biguanides (OR = 0.269, 95% CI = 0.112/0.610, *p* = 0.002), and statins (OR = 0.354, 95% CI = 0.159/0.761, *p* = 0.009) notably reduced MG-H1 levels (Table 4).

**Table 4:** Differences in the sensitivity of serum GL and MG-H1 levels to existing drug treatments

T2D	With *n*, (%)	GL			MG-H1			GL			MG-H1		
Oral administration		OR	95% CI (Lower/Upper)	*p*	OR	95% CI (Lower/Upper)	*p*	OR	95% CI (Lower/Upper)	*p*	OR	95% CI (Lower/Upper)	*p*
Glucose-independent insulin secretagogue	49 (32)	1.178	0.546/2.556	0.676	1.105	0.495/2.504	0.809						
Sulfonylurea	31 (20)							1.146	0.453/2.924	0.774	0.864	0.317/2.374	0.774
Glinide	18 (12)							1.323	0.459/3.940	0.605	1.791	0.571/6.084	0.329
Glucose-dependent insulin secretagogue	127 (83)	1.296	0.512/3.327	0.584	3.439	1.263/10.040	0.019∗						
DPP-4 inhibitor	100 (65)							1.253	0.489/3.245	0.638	2.465	0.938/6.764	0.072
GLP-1 receptor agonist	27 (18)							1.097	0.342/3.544	0.876	6.703	1.908/25.590	0.004∗∗
Carbohydrate absorption/excretion regulator	95 (62)	1.020	0.515/2.020	0.954	1.420	0.688/2.981	0.347						
α-Glucosidase inhibitor	23 (15)							0.849	0.334/2.133	0.726	1.777	0.670/4.942	0.255
SGLT2 inhibitor	83 (54)							1.137	0.593/2.186	0.698	1.353	0.680/2.718	0.391
Insulin sensitizer	78 (51)	0.916	0.443/1.887	0.811	0.462	0.210/0.989	0.049∗						
Biguanide	72 (47)							0.911	0.426/1.940	0.808	0.269	0.112/0.610	0.002∗∗
Thiazolidine	18 (12)							0.709	0.246/1.978	0.511	1.440	0.493/4.285	0.504
Insulin	25 (16)	1.708	0.694/4.364	0.250	2.749	1.016/8.081	0.053						
Statin	104 (68)	1.142	0.555/2.365	0.718	0.354	0.159/0.761	0.009∗∗						
Antihypertensive drug	89 (58)	1.593	0.828/3.093	0.165	1.446	0.722/2.913	0.299						
RAS inhibitor	65 (43)												
Calcium channel blocker	66 (43)												

The objective variable was discrete: 0 for groups of GL or MG-H1 levels below the median and one for groups above the median. The explanatory variable was discrete: 0 for patients without and one for patients with therapeutic drug. *n*, the number of patients with type 2 diabetes, who receive treatment with the therapeutic drug.

CI, confidence interval; DPP-4, dipeptidyl peptidase-4; GLP-1, glucagon-like peptide-1; OR, odds ratio; RAS, renin-angiotensin system; SGLT2, sodium glucose cotransporter-2; T2D, type 2 diabetes.

∗*p* < 0.05; ∗∗*p* < 0.01.

## Discussion

Early detection and therapeutic intervention are crucial for preventing diabetic complications; however, the existing markers of disease, including HbA1c, have several limitations. Therefore, the development of novel diabetic complication markers is highly desirable. Ideally, markers should be evaluable in easily collectible biological samples. However, a technique to measure GL from peripheral blood has not yet been established. As FL and GL have the same molecular weight, it is challenging to accurately measure GL using mass spectrometry (MS). In a previous study, we determined the stability of GL under acid hydrolysis conditions, when FL is completely converted to furosine, and reported the feasibility of GL identification using MS (25). However, there has not been confirmation in both *in vitro* and *in vivo* regarding the influence of FL on the measurement of GL in a state where GL and FL are mixed, as seen in biological samples (25). Therefore, the previous method was limited by insufficient detection sensitivity in peripheral blood. In this study, we aimed to establish a method for measuring GL with high sensitivity while avoiding cross-reactivity with FL. We first confirmed in the mixed solution of standard samples that the conversion of FL to furosine through acid hydrolysis does not impact the area value of GL (Fig. 1*A*). This was achieved by hydrolyzing samples with HCl after recovering fractions from the serum below a 3 kDa molecular weight (Fig. 1, *B*–*D*). Furthermore, in this study, we also demonstrated that the area value of *m/z* 309 changes upon acid hydrolysis of human serum (Fig. 1*B*). In general, in LC-MS/MS sample preparation for measuring AGEs, to prevent the artificial generation of AGEs from the precursors and intermediates during the preprocessing steps, a reduction step is applied before hydrolysis (39). In contrast, unlike CML, GL is not formed through the oxidation of Amadori products (25); thus, a reduction step is not required. Rather, if the serum samples undergo reduction, FL in the samples is converted to hexitol-lysine and is not converted to furosine (40, 41), rendering it difficult to distinguish it from GL. Considering these chemical characteristics, free GL in serum could be quantified by the conversion of FL to furosine and the resulting shift in molecular weight. However, it is not possible to quantify CML and GL simultaneously, since the former requires a reduction step before hydrolysis which would lead to GL not being indistinguishable. In the future, we intend to compare variations in various AGEs, including GL, and arrive at the appropriate conclusions then.

Helou *et al.* (42) found that dietary CML intake affects postprandial plasmatic free CML but not baseline, fasting free CML levels. Since our study uses fasting serum, the serum GL levels are assumed to be produced endogenously. Ketohexokinase, also known as fructokinase in the liver, which metabolizes fructose, has significantly higher activity compared to hexokinase and glucokinase, which metabolize glucose (43). Despite the transient increase in blood sugar concentration post high fructose solution ingestion, such as oral administration of corn syrup increases, its levels decline within an hour due to rapid liver uptake and metabolism. Even when fructose is administered intravenously, its half-life in blood is approximately half that of glucose (43). The rapid metabolism of fructose suggests that the measured values of serum fructose likely reflect its short-term concentrations. The fasting serum fructose level was 6.09 ± 0.57 μM in healthy individuals and 15.91 ± 6.83 μM in patients with type 2 diabetes (Fig. 2*A*), which is consistent with previous study findings (44). Previous research has shown that GL is produced only from fructose and not from other carbohydrates *in vitro* (25), but it is unclear if this can also be applied *in vivo*. Our study revealed a strong correlation between fructose and GL levels (Fig. 2*A*). However, the increase in the fructose level in patients with type 2 diabetes was 2.6-fold higher than that in healthy individuals, whereas the GL level showed an even greater increase—4.3-fold higher than that in healthy individuals (Fig. 2*B*). HbA1c is widely used as a clinical marker for diabetes because it reflects long-term blood glucose level fluctuations (45, 46), while glucose level reflects short-term changes. Similarly, fructose levels may reflect short-term fluctuations, while GL levels may reflect long-term fructose level fluctuations. This study suggests that further research on GL could clarify how fructose and GL levels fluctuate during different phases of diabetic complications.

Based on our experiments, when Schwann cells are cultured in high glucose, GL levels in both the culture medium and inside the cells increase by about 2.5-fold and 1.2-fold, respectively (Fig. 3, *A* and *B*). However, when Schwann cells were exposed to fructose at high levels similar to those found in the serum of patients with diabetic ketoacidosis or ketosis (47), the GL level in the culture medium and within the cells increased by about 1.4- and 1.1-fold, respectively, but there was no change under conditions of fructose level comparable to the blood level of healthy participants (Fig. 3, *C* and *D*). This finding suggests that even a temporary increase in the fructose level in the blood can have a marginal effect on GL production, and for GL to be generated *in vivo*, fructose must be continuously produced by the intracellular polyol pathway. Moreover, this study found that the increase in GL production owing to high glucose was significantly reduced when AR was knocked out (Fig. 3, *A* and *B*). This finding suggests that GL production is linked to the polyol pathway. We also observed that GL produced *via* the polyol pathway inside the cells is eventually excreted in the extracellular space (Fig. 3). Previous studies indicated that glucose itself does not significantly contribute to GL production (25), underscoring the importance of the polyol pathway enhancement over external fructose in GL production. Thus, this study not only presents the first report of GL level variations in human serum but also confirms the potential of GL as an indicator of the polyol pathway biochemically.

GL and MG-H1 levels in the serum of healthy participants and patients with type 2 diabetes exhibited different variation patterns. According to existing reports, an increase in free MG-H1 levels was observed in the serum of patients with type 2 diabetes (48). As such, here, we initially expected that the onset of type 2 diabetes would increase free MG-H1 levels. However, these decreased significantly, whereas free GL levels concomitantly increased with the development of diabetes (Fig. 4, *A* and *B*), one possible reason for this discrepancy is that previous studies detected free MG-H1 with a molecular weight of 10 kDa or less (48), while in our work, we quantified free MG-H1 with a molecular weight of 3 kDa or less. Additionally, as the therapeutic drugs took by patients with type 2 diabetes was also unknown, it could be possible that this did not include biguanides and/or statins, which were found to significantly decrease MG-H1 levels in this study (Table 4). Indeed, in patients with type 2 diabetes who are not receiving any drug treatment, the free MG-H1 levels in serum did not change (49). However, since they used a 0.2 μm pore size filter for pretreatment, the molecular weight range of free MG-H1 was unknown (49). Furthermore MG-H1 levels could have decreased, following a reduction in the amount of the precursor amino acid Arg. An existing report showed that, the amount of free Arg in the serum decreased with the onset of type 2 diabetes, and even more so with the development of additional complications (50). Our own findings corroborate this observation, as we found that serum Arg levels in patients with type 2 diabetes were significantly lower than those in healthy participants (Fig. 4*D*). Therefore, it is likely that free MG-H1 is significantly decreased, owing to the reduced Arg levels in the serum of patients with type 2 diabetes.

We evaluated the influence of vascular complications on fluctuations in GL and MG-H1 levels in patients with type 2 diabetes. GL levels increased with prolonged disease duration in type 2 diabetes patients (Table 2, Fig. S1). In addition, GL levels were lowest in healthy participants (Fig. 4*A*), followed by patients with type 2 diabetes without vascular complications, and the highest in patients with type 2 diabetes with vascular complications (Fig. 5, *A*, *D* and *G*). These results indicated a gradual increase in GL levels with disease severity. This observation was different from those of MG-H1, which increased only in patients with vascular complications (Figs. 4*B* and 5, *B*, *E* and *G*), and HbA1c, which increased in patients with type 2 diabetes (Table 1) but its levels were not affected by vascular complications (Fig. 5, *C*, *F* and *I*). This suggests that GL is suitabe for comparing the physiological states between all participants. Moreover, this study provides evidence that GL evaluations are important in patients with type 2 diabetes, because fluctuations in GL were consistently higher than those in MG-H1, FPG, HbA1c, and eGFR by the time of onset of type 2 diabetes and development of vascular complications (Table S1). Furthermore, we found that serum levels of both GL and MG-H1 were strongly influenced by eGFR (Table 2). This is speculated to be due to the increased retention of AGEs in the blood, resulting from the inability to excrete them due to decreased kidney function (51). Our data further showed that increases in GL were also strongly influenced by diabetes duration (Table 2). The risk factors for diabetes severity are primarily related to poor blood glucose control, it has been reported that diabetes duration is an important risk factor in microvascular complications (52). In addition, although the *p*-value was 0.043, GL was estimated to be an independent predictor of vascular complications, including microvascular complications in this study (Table 3). These findings indicate that the observed increase in GL levels is likely also attributed to factors other than kidney dysfunction, which is a strong risk factor for AGEs, unlike MG-H1. As a result, GL could be considered a candidate independent predictor of vascular complications.

The distinct difference between GL and MG-H1 lies in their differential sensitivity to drug treatments as well. Serum levels of AGEs may vary not only with the onset and progression of diabetes, but also depending on treatment interventions (53). In our work, we found that oral administration or not of any therapeutic drug, had a negligible effect on GL levels, but significantly altered MG-H1 levels in serum (Table 4). MG-H1 is generated by both insulin-dependent and insulin-independent glycolysis (16) and is considered highly sensitive to therapeutic drugs. In contrast, as GL is generated only from the polyol pathway in the insulin-independent pathway (26), it is likely less sensitive to therapeutic drugs. Therefore, it appears that GL may serve as a relatively stable predictor, less influenced by the drugs currently taken for the treatment of type 2 diabetes that focus on insulin secretion. In this context, an increase in MG-H1 levels due to GLP-1 receptor agonist treatment was observed in this study (Table 4). This is attributable to GLP-1 receptor agonist stimulating insulin secretion, leading to increased glucose uptake into cells and subsequent metabolism *via* glycolysis (54), consequently resulting in elevated blood levels of methylglyoxal, a precursor of MG-H1. Furthermore, decreased MG-H1 levels have been reported for patients with type 2 diabetes receiving biguanides (55), which we also observed in the present study (Table 4). This is attributed to the trapping of methylglyoxal by the biguanide (56, 57). Additionally, patients receiving statin treatment also reportedly present with decreased AGEs (58, 59), which we confirmed with MG-H1 in our work as well (Table 4). This is considered to be a pleiotropic effect of statins on adipokines (60). However, drugs that inhibit the removal or reabsorption of glucose in the blood such as α-glucosidase inhibitors and sodium glucose cotransporter-2 inhibitors did not affect AGEs levels. This finding suggests that AGEs produced inside cells may be released into the bloodstream. Our Schwann cell experiments support this notion on GL (Fig. 3). Additionally, the delayed release of sodium glucose cotransporter-2 inhibitors compared to other drugs, occurring in 2014, with short intake durations, may explain why there was no effect on AGEs levels. These findings suggest that conducting research focusing on drugs capable of inhibiting GL may lead to the development of new medications capable of inhibiting the progression of vascular complications caused by the activation of the polyol pathway.

Nevertheless, our study had certain limitations. First, this was a two-center cross-sectional study with a relatively small number of participants. As such, further large-scale prospective studies are needed to confirm the usefulness of serum GL and MG-H1 levels as predictors of vascular complications in patients with type 2 diabetes. Second, we only included Japanese patients. Further studies are therefore warranted on diverse populations, in order to examine the generalization of our current findings.

**Figure 6:**
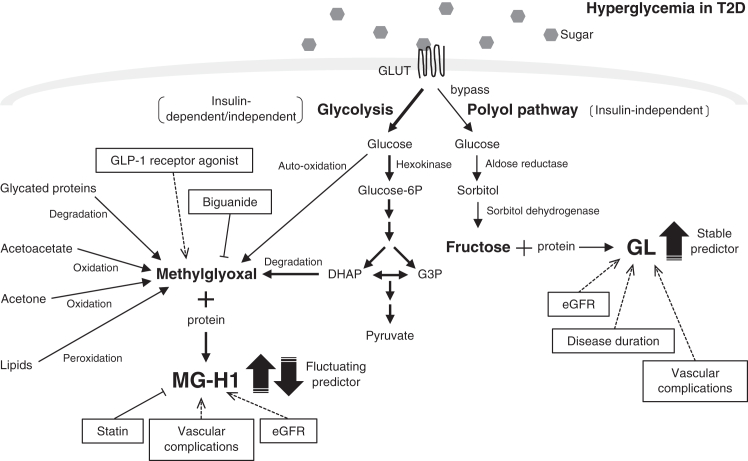
**Summary of fluctuations in glucose metabolism-related AGEs in type 2 diabetes.***Dashed arrows* represent promotion, while *T arrows* indicate inhibition. AGE, advanced glycation end-product; DHAP, dihydroxyacetone phosphate; eGFR, estimated glomerular filtration rate; G3P, glyceraldehyde 3-phosphate; GLP-1, glucagon-like peptide-1; glucose-6P, glucose 6-phosphate; GLUT, glucose transporter.

In conclusion, we presented a novel methodology for measuring GL, a glycation product of the polyol pathway, from serum. We demonstrated that the increase in GL levels indicates type 2 diabetes-associated vascular complications caused by enhanced polyol pathway activity. Also, GL could be a stable predictor independent of existing treatments (Fig. 6). Moreover, MG-H1 may indicate the metabolic status in patients with type 2 diabetes, particularly excessive glucose metabolism *via* glycolysis, but its changes depend on existing treatment (Fig. 6). The method we described in this study, which detects GL in peripheral blood rather than tissue, is versatile and can be used for various research purposes. We lay the groundwork for investigating the polyol pathway, including GL, and suggest new possibilities for drug development. Our study highlights the connection between polyol pathway activity and the development of vascular complications by focusing on GL, which is produced from fructose at the end of the polyol pathway, rather than relying on traditional indicators like AR activity or sorbitol content. Our results present valuable insights that could lead to novel and improved therapies for polyol pathway-related vascular complications.

## Experimental procedures

### Participants

To assess the utility of measuring GL levels in the serum of type 2 diabetes, the concentrations of GL and MG-H1, generated as a result of glucose metabolism, with various physiological parameters and the occurrence of complications were compared. We performed a cross-sectional study involving 177 patients with type 2 diabetes, recruited from outpatients at a diabetes clinic at the Kumamoto University Hospital (Kumamoto, Japan), between March 2021 and August 2021. Type 2 diabetes was diagnosed according to the World Health Organization criteria (61). Patients with type 1 diabetes, or patients testing positive for glutamic acid decarboxylase antibodies, or those with a history of ketoacidosis, or dependent on insulin therapy for survival, were excluded from the study. Patients with severe hepatic disease, malignancy, or acute/chronic inflammatory disease were also excluded (Fig. S4). Finally, data from 153 patients with type 2 diabetes (80 male, and 73 female) were analyzed. Preexisting use of glucose-lowering, antihypertensive, or antihyperlipidemic drugs was recorded. Moreover, healthy volunteers were recruited from among faculty and staff members of the Tokai University Kumamoto campus (Kumamoto, Japan) in December 2017. Participants with 5.7% or more HbA1c level, and 110 mg/dl or more of FPG, were excluded from the study. Finally, data from 21 participants (16 male and five female) were analyzed. The age, sex, height, weight, blood pressure, and smoking history of all patients were recorded. Body mass index was calculated as weight divided by height in meters squared (kg/m^2^).

### Ethics approval and consent to participate

All procedures involving human participants were performed in accordance with the ethical standards of the institutional and/or national research committee and the 1964 Helsinki Declaration and its later amendments, or comparable ethical standards. The study protocol involving patients was approved by the Human Ethics Review Committee of Kumamoto University (Protocol Number 1737) and registered at UMIN-CTR (UMIN000015966). When measuring the serum samples of these patients at Tokai University, the study was approved by the Human Ethics Review Committee of Tokai University (Approval Number 21179). The study protocol for healthy volunteers was approved by the Human Ethics Review Committee of Tokai University (Approval Number 17070). All participants provided written informed consent.

### Measurement of blood parameters

To avoid the influence of external factors on glucose homeostasis and dietary AGEs, morning blood samples were collected from fasted participants. The total cholesterol, triglyceride, high-density lipoprotein cholesterol, FPG, and HbA1c were measured using a Hitachi 7600 analyzer (Hitachi Ltd). The low-density lipoprotein cholesterol concentration was determined using the Friedewald formula (62), while the eGFR was calculated using the formula recommended by the Japanese Society of Nephrology (63, 64). High sensitivity C-reactive protein (hsCRP) concentration was measured using a latex aggregation method (Denka Seiken Co, Ltd) on a BM2250. The fasting plasma insulin (FPI) concentration was determined using an electrochemiluminescence method (Roche Diagnostics K.K.) on a Modular Analytics E module. Insulin resistance was assessed using homeostatic model assessment-insulin resistance (HOMA-IR), and was calculated using the following formula:

HOMA-IR = FPI (μU/ml) × FPG (mmol/L)/22.5 (65).

The C-peptide index (CPI), which represents endogenous insulin secretion, was calculated as follows:

CPI = hsCRP (ng/ml)/FPG (mg/dl) × 100.

Patients with microvascular complications were defined as those having diabetic retinopathy, neuropathy, or nephropathy. Patients with macrovascular complications were defined as those having coronary artery disease, cerebrovascular disease, or peripheral artery disease. Patients with vascular complications were defined as those having diabetic microvascular and/or macrovascular complications.

### Chemicals

The standards of GL and FL (25), and MG-H1 (66), were synthesized as previously described. Lys and Arg were purchased from Fuji Film Wako Pure Chemical. The isotope-labeled internal standards of [^13^C_6_] GL and [^13^C_6_] FL, were prepared as previously described (25), while [^2^H_3_] MG-H1 was purchased from PolyPeptide Laboratories. Similarly, [^13^C_6_] Lys and [^13^C_6_] Arg were procured from Cambridge Isotope Laboratories Inc. D-Fructose was purchased from Kanto Chemical. D-[^13^C_6_] Fructose was procured from Omicron Biochemicals. All other chemicals used were of the highest commercial grade.

### Preparation for confirmation with a standard mix of GL and FL

The GL and/or FL from standards and internal standards were added at a final concentration of 10 pmol. Subsequent procedures were performed in the same manner as for serum samples and cell samples (described later).

### Preparation of serum sample for measurement of free GL, MG-H1, Lys, and Arg

Human serum (250 μl) was filtered through a 3 K molecular weight cut-off (MWCO) filter (VIVASPIN 500; Sartorius AG) at 10,000*g*, 15 °C for 1 h. We next added 10 μl of 1 μM [^13^C_6_] GL and [^2^H_3_] MG-H1, as well as 5 μl of 1 mM [^13^C_6_] Lys and [^13^C_6_] Arg, to 100 μl of filtrate. The samples were hydrolyzed with 1 ml of 6 M HCl at 100 °C for 18 h. Following hydrolysis, the samples were dried *in vacuo*.

### Cell culture

Spontaneously immortalized mouse Schwann cells of WT (IWARS1) and AR KO (IKARS1) were prepared as previously described (35, 67). The cells were cultured in Dulbecco’s modified Eagle’s medium (DMEM; Fuji Film Wako Pure Chemical) containing 5% fetal bovine serum (FBS; Biosera), 20 ng/ml neuregulin-β (Recombinant Human NRG1-β/HRG1-β EGF Domain, Carrier-free; R&D systems Inc), 100 μg/ml penicillin (Meiji Seika Pharma), and 100 units/ml streptomycin (Meiji Seika Pharma). The cells were maintained in a humidified incubator with 5% CO_2_ at 37 °C.

### Preparation for measurement of GL contents in Schwann cells and culture medium under high sugar conditions

IWARS1 and IKARS1 cells suspended in DMEM containing 5% FBS and no neuregulin-β were seeded in a 6-well plate. After preculture for 20 h, the cells were starved in DMEM containing 1% FBS medium for 4 h and then for 3 days were incubated in 5.5 mM glucose medium and supplemented with 45 mM mannitol for osmotic adjustment, 45 mM glucose, and 10 or 100 μM fructose. Subsequently, the cells were rinsed twice with ice-cold D-PBS, and lysed in 1% CHAPS on ice for 30 min. The lysed cells were collected using a cell scraper and placed in an ultrasonic disruptor UD-211 (Tomy Seiko Co, Ltd) for 5 s on ice. The cell debris was removed from each sample by centrifugation at 10,000*g* and 4 °C for 5 min and then the supernatants were collected. At the same time, the cultured mediums were also collected, centrifuged at 200*g* and 4 °C for 5 min, and filtered through a 0.45 μm filter composed of hydrophilic polytetrafluoroethylene (Tomsic Ltd). These samples were stored at −80 °C until use.

The AGEs contents in the cells and cultured medium samples were measured using LC-MS/MS as previously described (68), with minor modifications. Briefly, the cells samples (50 μg) were precipitated with an equal volume of 20% trichloroacetic acid at 4 °C for 30 min. Then, 150 μl of 6 M HCl was added to the pellets and were heated at 100 °C for 1 h to dissolve. Thereafter, internal standard and 850 μl of 6 M HCl was added to the protein samples, and they were hydrolyzed at 100 °C for 18 h. The cultured medium samples were filtered through a 3 K MWCO filter at 10,000*g* and 15 °C for 1 h. Internal standard and 1 ml of 6 M HCl were added to the filtrates (200 μl), and they were hydrolyzed at 100 °C for 18 h. Following hydrolysis, the samples were dried *in vacuo*.

### Solid-phase extraction of sample preparation for LC-MS/MS using a fully automated solid-phased extraction system

Solid-phase extraction was performed by fully automated solid-phased extraction system (Shimadzu Corporation), as previously described (69). Briefly, dried samples were resuspended in 1 ml of 0.1% (v/v) trifluoroacetic acid (TFA). The samples (950 μl) were passed over the cation-exchange column (Strata-X-C; Phenomenex), which had been prewashed with 1 ml of methanol and equilibrated with 1 ml of 0.1% (v/v) TFA. The column was washed with 3 ml of 2% (v/v) formic acid (FA) and eluted with 1 ml of 7% (v/v) ammonia. The pooled elution fractions were air-dried.

### Measurement of GL, MG-H1, Lys, and Arg levels by LC-MS/MS

Free AGEs and free amino acid levels in the serum samples were measured using LC-MS/MS as previously described (25, 39, 66), with minor modifications. Briefly, the air-dried samples were resuspended in 20% (v/v) acetonitrile containing 0.1% (v/v) FA in 250 μl for cell samples and 500 μl for medium samples; the standard samples and serum samples volume were 1 ml. The samples were filtered through a 0.45 μm filter of hydrophilic polytetrafluoroethylene. Next, samples (10 μl) were subjected to LC-MS/MS. The LC was conducted using an LC-40 system (Shimadzu Corporation), and separated on a hydrophilic interaction chromatography column (150 × 2.1 mm, 5 μm) (ZIC-HILIC; Merck Millipore). The flow rate was set at 0.2 ml/min, and the column was maintained at 40 °C. The mobile phase consisted of solvent A (distilled water containing 0.1% (v/v) FA) and B (acetonitrile containing 0.1% (v/v) FA). The gradient profile was as follows: 90% B (0–2 min), 90–10% B (2–16 min), and 10% B (16–19 min). The retention times of GL, MG-H1, Lys, and Arg were 12 to 14 min. The MS was conducted using an LCMS-8060NX (Shimadzu Corporation), operated in the positive ion mode using the following parameters of the interface in ion-focus: nebulizer gas inflow, 3 L/min; heating gas inflow, 15 L/min; interface temperature, 400 °C; DL temperature, 250 °C; heat-block temperature, 400 °C; drying gas inflow 3 L/min. The precursor and product ions of the analytes are shown in Table 5, along with the corresponding Q1 precursor bias, collision energy, and Q3 precursor bias. Data acquisition and analysis were performed using the LabSolutions software (Shimadzu Corporation, https://www.an.shimadzu.co.jp/products/software-informatics/intro/index.html). The samples were quantified by an internal standard method, utilizing standard curves constructed from mixtures of isotopically labeled and nonlabeled standards.

**Table 5:** The *m/z* values of GL, MG-H1 and amino acids, based on LC-MS/MS analysis

Analyte	Polarity	Precursor ion (*m/z*)	Product ion (*m/z*)	Q1 pre bias (V)	Collision energy	Q3 pre bias (V)
GL	Positive	309.2	84.1	−21	−35	−30
[^13^C_6_] GL	Positive	315.2	84.1	−15	−14	−15
MG-H1	Positive	229.2	114.1	−12	−16	−19
[^2^H_3_] MG-H1	Positive	232.2	117.1	−16	−16	−22
Lys	Positive	147.1	84.1	−11	−40	−29
[^13^C_6_] Lys	Positive	153.2	89.1	−11	−35	−14
Arg	Positive	175.1	70.2	−13	−5	−27
[^13^C_6_] Arg	Positive	181.2	74.2	−13	−10	−27

Abbreviation: LC-MS/MS, liquid chromatography-tandem mass spectrometry.

### Measurement of fructose levels in the serum by LC-quadrupole(Q)TOF

A mixture of human serum (100 μl) and 5 nmol [^13^C_6_] fructose dissolved in 1% (v/v) TFA (0.5 ml) was passed through a 3 K MWCO filter at 12,000*g* for 45 min at 4 °C. The filtrate (100 μl) was then mixed with 1% (v/v) TFA (900 μl) and filtered through the C18 column (Sep-pak Vac 1 cc C18 Cartridges; Waters Corporation), which had been prewashed with methanol (1 ml) and equilibrated with 1% (v/v) TFA (1 ml). The pooled flow-through fractions and 0.4 ml of the same solution fractions were passed over the cation-exchange column (Strata-X-C), which was prewashed with 1 ml of methanol and equilibrated with 1 ml of distilled water. The flow-through fractions and 0.4 ml of the same solution fractions were pooled and air-dried. The dried samples were resuspended in 0.1 ml of 20% (v/v) acetonitrile containing 10 mM ammonia. The fructose levels in the sample was measured by LC-quadrupole time-of-flight (Bruker Daltonics). LC was conducted on a ZIC-HILIC column maintained at 60 °C. The mobile phase consisted of solvent A (10 mM ammonia solution) and B (acetonitrile). The gradient pattern was as follows: 95% B (0–5 min), 90–80% B (5–15 min), and 80% B (15–20 min). The flow rate was set to 0.3 ml/min and the injection volume was 3 μl. Fructose and [^13^C_6_] fructose were detected using MS (negative). The extracted ions of fructose and [^13^C_6_] fructose were at *m/z* 179.0550 (±0.01) and *m/z* 185.0751 (±0.005), respectively. The retention time of fructose and its internal standard was approximately 12 min.

### Statistical analysis

Details of the statistical analysis were provided in the Supporting information. All statistical analyses were performed using EZR version 4.2.2 (https://www.jichi.ac.jp/saitama-sct/SaitamaHP.files/statmed.htmlR https://posit.co/download/rstudio-desktop/) for Mac (Saitama Medical Center, Jichi Medical University, Saitama, Japan), a graphical user interface for R (The R Foundation for Statistical Computing, Vienna, Austria) (70). A *p*-value <0.05 was considered as statistically significant.

## Data availability

The datasets used and/or analyzed during the current study are available from the corresponding author on reasonable request.

## Supporting information

This article contains supporting information (70, 71, 72, 73).

## Conflict of interest

The authors declare that they have no conflicts of interest with the contents of this article.
