# Supplementary material for: Glucoselysine, a unique advanced glycation end-product of the polyol pathway and its association with vascular complications in type 2 diabetes
Source: J Biol Chem. 2024 Jun 13;300(7):107479. doi: 10.1016/j.jbc.2024.107479 (PMC11283207; doi:10.1016/j.jbc.2024.107479)
Supplement: Supporting Information [file mmc1.docx]

**Supporting information**

**Glucoselysine, a unique advanced glycation end-product of the polyol pathway and its association with vascular complications in type 2 diabetes**

Hiroko Yamaguchi^1^, Takeshi Matsumura^2^, Hikari Sugawa^3^, Naoko Niimi^4^, Kazunori Sango^4^, Ryoji Nagai^1,3^*

^1^Laboratory of Food and Regulation Biology, Graduate School of Bioscience, Tokai University, Kumamoto, Japan

^2^Department of Metabolic Medicine, Faculty of Life Sciences, Kumamoto University, Kumamoto, Japan

^3^Laboratory of Food and Regulation Biology, Department of Food and Life Science, School of Agriculture, Tokai University, Kumamoto, Japan

^4^Diabetic Neuropathy Project, Department of Diseases and Infection, Tokyo Metropolitan Institute of Medical Science, Tokyo, Japan

*Corresponding author: Ryoji Nagai, Ph.D.

Laboratory of Food and Regulation Biology, Department of Food and Life Sciences, School of Agriculture, Tokai University, Sugidoh 871-12, Mashiki-machi, Kamimashiki-Gun, Kumamoto 861-2205, Japan.

TEL: +81-96-386-2692

E-Mail: [nagai-883@umin.ac.jp](about:blank)

**Running title:** Glucoselysine associated with diabetic complications

**This file includes:** Table S1, Fig. S1 to S4, Experimental procedures

**Table S1 Degree of change in healthy participants and patients with type 2 diabetes**

|  | T2D | | MICRO | | MACRO | | VCOMP | |
| --- | --- | --- | --- | --- | --- | --- | --- | --- |
|  | (fold) | (%) | (fold) | (%) | (fold) | (%) | (fold) | (%) |
| GL | 1.5 | 34.0 | 1.4 | 28.6 | 1.3 | 21.0 | 1.5 | 34.0 |
| MG-H1 | 0.8 | -20.1 | 1.1 | 9.5 | 1.2 | 15.1 | 1.1 | 12.2 |
| FPG | 1.6 | 38.8 | 1.2 | 17.6 | 1.2 | 16.1 | 1.2 | 19.7 |
| HbA1c | 1.4 | 29.2 | 1.0 | 2.9 | 1.0 | 3.6 | 1.0 | 3.6 |
| eGFR | 0.9 | -8.1 | 0.8 | -8.2 | 0.8 | -24.2 | 0.9 | -10.1 |

Degree of changes was calculated relative to patients without the respective disease. The mean values were utilized for these calculations. T2D, type 2 diabetes; MICRO, microvascular complications; MACRO, macrovascular complications; VCOMP, micro and/or macrovascular complications; FPS, fasting plasma glucose; HbA1c, hemoglobin A1c; eGFR, estimated glomerular filtration rate.

**Fig. S1 Correlation between GL, MG-H1, and HbA1c levels and clinical parameters in type 2 diabetes patients.**

Correlation between GL and duration of type 2 diabetes (a), GL and eGFR (b), MG-H1 and eGFR (c), and HbA1c and FPG (d). eGFR, estimated glomerular filtration rate; FPG, fasting plasma glucose; *r*, Pearson product-moment correlation coefficient.

**Fig. S2 Association between GL and MG-H1 levels and microvascular complications in patients with type 2 diabetes.**

Levels of GL in retinopathy (DR) (a), neuropathy (DP) (b), and nephropathy (DN) (c) in patients with type 2 diabetes. Levels of MG-H1 in DR (d), DP (e), and DN (f) in patients with type 2 diabetes. Patients with type 2 diabetes without (*n* = 112) or with (*n* = 41) DR, without (*n* = 115) or with (*n* = 38) DP, without (*n* = 87) or with (*n* = 66) DN. The concentration of the internal standard in the human serum was 10 pmol. Welch's two-sample *t* test, **p*<0.05, ***p*<0.01.

**Fig. S3 Association between GL and MG-H1 levels and macrovascular complications in patients with type 2 diabetes.**

Levels of GL in coronary artery disease (CAD) (a), cerebral vascular disease (CVD) (b), and peripheral artery disease (PAD) (c) in patients with type 2 diabetes. Levels of MG-H1 CAD (d), CVD (e), and PAD (f) in patients with type 2 diabetes. Patients with type 2 diabetes without (*n* = 142) or with (*n* = 11) CAD, without (*n* = 130) or with (*n* = 23) CVD, and without (*n* = 143) or with (dense dotted box, *n* = 10) PAD. The concentration of the internal standard in the human serum was 10 pmol. Welch's two-sample *t* test, **p*<0.05, ***p*<0.01.

**Fig. S4 Flow chart illustrating the patients with type 2 diabetes enrollment process.**

T2D, type 2 diabetes; T1D, type 1 diabetes.

**Experimental procedures**

**Assessment of diabetic microvascular complications**

The urinary albumin-to-creatinine ratio (ACR) was calculated using urinary albumin and creatinine concentrations measured in early morning fasting spot urine samples. According to the criteria of the Japan Diabetes Society (72), diabetic nephropathy was diagnosed based on the estimated glomerular filtration rate (eGFR) and the presence of albuminuria or proteinuria as follows:

ACR ≥30 mg/gCr or eGFR <30 mL/min/1.73 m^2^

Patients who underwent dialysis were excluded. Diabetic retinopathy was defined as the presence of characteristic changes, such as microaneurysms, hemorrhages, cotton wool spots, and new vessel formation. Patients who had previously received treatment for diabetic retinopathy, such as laser photocoagulation or vitreous surgery, were also defined as having diabetic retinopathy. All patients were diagnosed by ophthalmologists. The presence of diabetic neuropathy was evaluated using the abbreviated diagnostic criteria proposed by the Diabetic Neuropathy Study Group in Japan (73). The criteria consisted of three items: (i) symptoms such as tingling pain, numbness, and cramping; (ii) absence of the Achilles tendon reflex; and (iii) reduced sense of vibration. Patients with more than two positive symptoms were defined as having diabetic neuropathy.

**Statistical analysis**

For the mixture of the hydrolyzed glucoselysine (GL) and fructoselysine (FL) standard products, the respective ratios were compared using Dunnett test, with a mixture of GL and [^13^C_6_] GL as the reference. The differences in the GL peak area between healthy participants and patients with type 2 diabetes based on the presence or absence of hydrolysis, were assessed using a paired *t* test and Welch's two-sample *t* test following logarithmic transformation. The GL quantification values among healthy participants were logarithmically transformed and compared using one-way ANOVA with Bonferroni correction.

The correlation between serum fructose levels and GL levels was analyzed using Spearman's rank correlation coefficient. Additionally, the degree of change in patients with type 2 diabetes calculated relative to healthy participants, with the comparison of variation values was expressed as the mean ± standard deviation and assessed using the Mann-Whitney U test.

All Schwann cell experiment results are expressed as the mean ± standard deviation and were compared using one-way ANOVA with Bonferroni correction.

All clinical characteristics were expressed as the median (25th-75th percentile) and compared using Welch's two-sample *t* test. The values of the metabolites in the blood are presented as boxplots. Statistical tests between the groups were performed using Welch’s two-sample *t* test after logarithmically transforming the values.

Correlation coefficients were analyzed using Pearson’s product-moment correlation coefficient. Multiple regression analysis was performed using data from 153 cases of GL and *N*^δ^-(5-hydro-5-methyl-4-imidazolon-2-yl)ornithine (MG-H1), and 151 cases of hemoglobin A1c (HbA1c). The GL, MG-H1, and HbA1c were objective variables of continuous numerical values, while age, sex, body mass index, diabetes duration, FPG, and HbA1c were selected as explanatory variables based on previous studies. Other explanatory variables were appropriately selected from factors that correlated in univariate analysis. A binomial logistic regression analysis was performed using data from 153 cases. For the analysis on diabetic complications, micro- or macrovascular complications were the objective variables of the binary variable: 0 for patients without vascular complications and 1 for patients with vascular complications. The explanatory variables GL, MG-H1, and HbA1c were continuous. To conduct the binomial logistic analysis appropriately, the GL and MG-H1 values were scaled by 10 before calculation (74). For the analysis on therapeutic drugs, GL or MG-H1 levels were the objective variables of the binary variable: 0 for groups with GL or MG-H1 levels below the median and 1 for groups with levels above the median. The explanatory variable was discrete: 0 for patients without therapeutic drugs and 1 for patients with therapeutic drugs. A primary analysis was conducted to verify any changes induced by therapeutic drugs categorized into seven groups: glucose-independent insulin secretagogues, glucose-dependent insulin secretagogues, carbohydrate absorption/excretion regulators, insulin sensitizers, insulin preparations, antihyperlipidemic drugs, and antihypertensive drugs. This was followed by a secondary analysis focused on glucose-lowering drugs.

All figures and tables were drawn in Word and Excel using Microsoft Office for Mac version 16.76. All statistical analyses were performed using EZR version 4.2.2 for Mac (Saitama Medical Center, Jichi Medical University, Saitama, Japan), a graphical user interface for R (The R Foundation for Statistical Computing, Vienna, Austria) (71). A *p*-value <0.05 was considered as statistically significant.
